# Early use of long‐acting injectable antipsychotics in bipolar disorder type I: An expert consensus

**DOI:** 10.1111/bdi.13498

**Published:** 2024-10-22

**Authors:** Eduard Vieta, Mauricio Tohen, Diane McIntosh, Lars Vedel Kessing, Martha Sajatovic, Roger S. McIntyre

**Affiliations:** ^1^ Hospital Clinic, Institute of Neuroscience, University of Barcelona, IDIBAPS, CIBERSAM Barcelona Spain; ^2^ Department of Psychiatry and Behavioral Sciences University of New Mexico Health Science Center Albuquerque New Mexico USA; ^3^ Department of Psychiatry University of British Columbia Vancouver British Columbia Canada; ^4^ Copenhagen Affective Disorder Research Center (CADIC) Psychiatric Center Copenhagen, Frederiksberg Copenhagen Denmark; ^5^ Department of Clinical Medicine, Faculty of Health and Medical Sciences University of Copenhagen Copenhagen Denmark; ^6^ Department of Psychiatry University Hospitals Cleveland Medical Center Cleveland Ohio USA; ^7^ Department of Neurology University Hospitals Cleveland Medical Center Cleveland Ohio USA; ^8^ Neurological and Behavioral Outcomes Center University Hospitals Cleveland Medical Center Cleveland Ohio USA; ^9^ Case Western Reserve University School of Medicine Cleveland Ohio USA; ^10^ University of Toronto Toronto Ontario Canada; ^11^ Brain and Cognition Discovery Foundation Toronto Ontario Canada

**Keywords:** antipsychotics, bipolar disorder type 1, consensus, patient‐reported outcome measures, psychosis, shared decision‐making, treatment adherence and compliance

## Abstract

**Introduction:**

Long‐acting injectable antipsychotics (LAIs) are not routinely offered to patients living with bipolar disorder type I (BP‐I), despite widespread evidence that supports their benefits over oral antipsychotics, particularly in early disease.

**Methods:**

A round‐table meeting of psychiatrists convened to discuss barriers and opportunities and provide consensus recommendations around the early use of LAIs for BP‐I.

**Results:**

LAIs are rarely prescribed to treat BP‐I unless a patient has severe symptoms, sub‐optimal adherence to oral antipsychotics, or has experienced multiple relapses. Beyond country‐specific accessibility issues (e.g., healthcare infrastructure and availability/approval status), primary barriers to the effective use of LAIs were identified as attitudinal and knowledge/experience‐based. Direct discussions between healthcare providers and patients about treatment preferences may not occur due to a preconceived notion that patients prefer oral antipsychotics. Moreover, as LAIs have historically been limited to the treatment of schizophrenia and the most severe cases of BP‐I, healthcare providers might be unaware of the benefits LAIs provide in the overall management of BP‐I. Improved treatment adherence associated with LAIs compared to oral antipsychotics may support improved outcomes for patients (e.g., reduced relapse and hospitalization). Involvement of all stakeholders (healthcare providers, patients, and their supporters) participating in the patient journey is critical in early and shared decision‐making processes. Clinical and database studies could potentially bridge knowledge gaps to facilitate acceptance of LAIs.

**Conclusion:**

This review discusses the benefits of LAIs in the management of BP‐I and identifies barriers to use, while providing expert consensus recommendations for potential solutions to support informed treatment decision‐making.

## 1 INTRODUCTION

Bipolar disorder is a severe and chronic mental disorder characterized by fluctuations in mood between abnormally elevated highs (known as mania or hypomania episodes) and emotional lows (known as depressive episodes), affecting approximately 45 million patients worldwide.^1^ Although the severity of symptoms varies from person to person, manic and hypomanic episodes largely share the same symptoms, which can include an exaggerated sense of self‐esteem, impulsive or reckless behavior, racing thoughts, and a decreased need for sleep.^2^ Depressive episodes involve symptoms, such as intense sadness or despair, changes in appetite or sleep, recurrent thoughts of death or suicide, feelings of guilt and agitation, difficulty concentrating, and reduced energy or fatigue.^3^ Psychosis (i.e., the occurrence of hallucinations or delusions) is also a common symptom of bipolar disorder; more than half of patients living with bipolar disorder have a lifetime history of psychotic symptoms,^4^ and the presence of psychotic symptoms in bipolar disorder has been associated with poorer patient outcomes.^5^ Bipolar disorder is often first diagnosed in late adolescence or early adulthood after several years of symptoms, varying greatly from person to person, thus making it a difficult disorder to diagnose.^6^, ^7^

Patients living with bipolar disorder report poor quality of life and severe role impairment, impacting on their ability to function, maintain relationships and stable employment, and make sound judgments.^8^, ^9^, ^10^, ^11^, ^12^ Compared to patients without the condition, bipolar disorder is associated with more missed workdays annually on average,^13^, ^14^ and many employees living with the disorder end up on short‐term disability due to the high number of missed workdays.^12^, ^14^

Bipolar disorder type I (BP‐I) is characterized by manic or mixed episodes, with or without depression, with a lifetime prevalence of 0.6%.^8^ Mania is the defining feature of BP‐I.^15^ As mentioned, diagnosis and treatment of bipolar disorder, including BP‐I, is challenging; of note, increasing evidence is available on mania without major depressive episodes, also known as unipolar mania, which may represent a distinct diagnostic condition.^16^

Course specifiers are extensions to a diagnosis that further clarify the course, severity, or special features of the disorder.^17^ Increasing evidence suggests that the concept of predominant polarity, a proposed course specifier for bipolar disorder, may have clinical relevance for the management of bipolar disorder,^18^ which is closer to the real‐world clinical practice and more pragmatic than the nosologically complex differentiation between bipolar subtypes such as BP‐I and bipolar disorder type II (BP‐II).^19^ For optimal outcomes, bipolar disorder requires an individualized, long‐term, patient care management plan, and an integrative approach that includes maintenance treatment, adjunctive psychosocial therapies, diligent monitoring for any treatment‐emergent complications, and promotion of a healthy lifestyle including stress management.^20^, ^21^, ^22^

Accurate and timely diagnosis, prompt implementation of effective therapies, and medication adherence remain some of the key challenges and unmet needs in the management of bipolar disorder.^23^ Non‐adherence to treatment is associated with significantly increased risks of recurrence, relapse, hospitalization, and suicide attempts, and higher overall treatment costs.^24^ The estimated direct and indirect costs of bipolar disorder, including BP‐I, in the United States alone are estimated to be more than $195 billion annually,^25^ yet the greatest societal cost associated with bipolar disorder relates to significant loss of life due to suicide,^26^ followed by cardiovascular disease and other physical comorbidities.^27^, ^28^ Furthermore, bipolar disorder can cause progressive brain damage; thus, frequent relapses may result in greater cognitive and functional impairment, increasing the burden of illness for the patients and their supporters.^29^, ^30^, ^31^, ^32^

For several decades, lithium has been, and remains, the “gold standard” first‐line treatment for patients living with BP‐I, followed by other oral medications (such as olanzapine, quetiapine, and risperidone orally disintegrating tablet).^33^, ^34^, ^35^ Despite declining prescription rates during the past decade due to the adverse‐event and toxicity burden, it is used because of its effectiveness in both BP‐I and BP‐II, including for the prevention of suicidal behavior.^36^, ^37^, ^38^ In recent years, LAIs such as aripiprazole and risperidone have emerged as potential maintenance treatment options for patients living with BP‐I,^21^ including early in the course of the illness.^39^, ^40^, ^41^, ^42^ At present, aripiprazole and risperidone are approved by the US Food and Drug Administration; however, the use of LAIs for bipolar disorder is currently off‐label in Europe according to European Medicines Agency recommendations.^43^ Compared to oral antipsychotics that are shorter acting and must be taken more frequently, LAIs are designed to provide relief of symptoms by slowly releasing the drug over an extended period (weeks to months).^44^ Studies in schizophrenia and bipolar disorder suggest that LAIs may provide additional benefits over oral antipsychotics by reducing the rates of re‐hospitalization and risk of relapse, improving overall quality of life, and improving adherence to treatment.^41^, ^45^, ^46^, ^47^ However, LAIs are not routinely offered to patients living with BP‐I,^48^ for reasons that will be explored in this expert consensus report.

The main objectives of this article are to highlight the potential benefits of LAIs in the management of BP‐I, particularly in early disease, and to discuss the many barriers faced during implementation, while providing expert consensus recommendations for potential solutions.

## 2 METHODS

A round‐table meeting of expert psychiatrist advisors was convened online to discuss the barriers and opportunities for early use of LAIs for the treatment of patients living with BP‐I. Following an in‐depth discussion of key disease‐state topics and a review of current clinical practice around BP‐I and LAIs, the advisors discussed recommendations related to the early use of LAIs in BP‐I.

## 3 RESULTS AND DISCUSSION

### 3.1 BP‐I and LAIs

Currently, LAIs are rarely prescribed for the treatment of people living with BP‐I.^48^ According to the advisors, key characteristics of a patient typically offered an LAI include very severe symptoms, sub‐optimal adherence to orally administered antipsychotics, and having experienced multiple relapses. Historically, LAI use was limited to the treatment of patients with schizophrenia, while for BP‐I, LAIs were employed as a treatment of “last resort.” This may be due to healthcare providers (HCPs) perceiving BP‐I as a “milder” disorder in comparison to schizophrenia and having a preconceived (yet unsubstantiated) notion of a patient's preference for oral antipsychotics. These opinions may stem from an overall lack of awareness of the benefits of LAIs: LAIs have only recently begun to be indicated for the treatment of patients living with BP‐I and have been slow to appear in major guidelines.^34^, ^49^ In addition to the lack of awareness, the limited presence in guidelines presents further barriers to use, including issues with healthcare reimbursement.

However, there is mounting evidence to support LAI use in the treatment of patients living with BP‐I, particularly in early disease (Table 1),^41^, ^42^, ^50^, ^51^, ^52^ with worldwide studies demonstrating that treatment with LAIs, such as flupenthixol and risperidone, early in the disease course was well tolerated and resulted in better outcomes compared to oral medications (such as olanzapine and quetiapine), including relapse prevention.^51^, ^52^ A recent review of evidence from six mirror‐image studies of LAIs for the treatment of bipolar disorder emphasizes that these therapies may be an effective strategy to improve major clinical outcomes.^53^ LAI treatment was associated with a significant reduction in hospitalizations and a lower number of emergency department visits in the year after LAI initiation. In addition, a significant decrease in hypomanic/manic relapses after LAI treatment initiation was observed, while the effect of LAIs for depressive episodes was less clear. Of note, there are recent observational data from a real‐world effectiveness nationwide cohort study in Finland supporting LAIs (haloperidol, perphenazine, risperidone, and zuclopenthixol) in reducing the risk of psychiatric and all‐cause re‐hospitalization compared to oral antipsychotic counterparts,^41^ indirectly emphasizing the relevance of treatment adherence.^54^ According to those studies, the prevention of hospitalization may be due to the mood‐stabilizing capability of LAIs.^55^

**TABLE 1:** Overview of available data supporting the use of LAIs in bipolar disorder (including BP‐I).

Study details/publication type	Location	Intervention/treatment	Main finding
*Bipolar disorder* (*including BP*‐*I*)			
Real‐world effectiveness nationwide cohort study^41^	Finland	Lithium (oral) and several other oral medications vs. LAIs (haloperidol, perphenazine, risperidone, zuclopenthixol)	LAIs significantly reduced the risk of psychiatric and all‐cause re‐hospitalization compared to oral antipsychotic counterparts
Comprehensive literature review^42^	Worldwide	Lithium, olanzapine, divalproex, psychological treatment	Treatment in earlier‐phase illness resulted in better outcomes, including relapse rate and psychosocial functioning
Systematic review and meta‐analyses of RCTs^52^	Worldwide	Flupenthixol, risperidone, various oral medications (mood stabilizer, antidepressant, antipsychotic, or any combination of these agents)	LAIs were beneficial for relapse prevention in patients with rapid cycling
Literature review on efficacy and safety of first‐ and second‐generation LAIs for maintenance treatment of bipolar disorder^51^	Worldwide	First‐generation LAIs, including flupenthixol, haloperidol, fluphenazine, and a mix of diverse antipsychotics, and second‐generation LAI risperidone	Risperidone was effective as maintenance treatment in bipolar disorder, notably for preventing mania with no worsening in depression, whereas first‐generation LAIs possibly increased the risk of worsening depression. Risperidone was also better tolerated than first‐generation LAIs
*Early‐phase psychosis* (*e.g*., *schizophrenia or bipolar disorder*)			
Systematic review, meta‐analysis, and meta‐regression^50^	Worldwide	10 RCTs comparing EIS with TAU	Early psychological and pharmacologic intervention treatments resulted in better outcomes compared to TAU

Abbreviations: BP‐I, bipolar disorder type I; EIS, early intervention services; LAI, long‐acting injectable antipsychotic; RCT, randomized clinical trial; TAU, treatment as usual.

Four‐year prospective data from the Systematic Treatment Optimization Program have shown that best practices (e.g., safe and effective maintenance treatment regimens, psycho‐education, cognitive behavioral therapy, interpersonal and social rhythm therapy, family‐focused therapy, and peer‐support and stress‐management programs) in the treatment of first‐episode mania in patients living with BP‐I resulted in remission and recovery. Additionally, while recurrence was common, minimizing recurrence within the first year through risk‐factor modification (e.g., reducing anxiety, stress, and comorbid alcohol/substance abuse) may alter the course of the disease.^56^ Therefore, timely intervention with LAIs represents a critical opportunity for HCPs to engage in shared decision‐making with the patient and their supporters, helping them make informed decisions about treatments and setting the wheels in motion for positive long‐term outcomes, including the prevention of key disease concerns such as relapse.^57^

LAIs have shown better efficacy in preventing mania than depression in patients living with bipolar disorder^58^, ^59^; therefore, LAIs may be a useful first‐line treatment for patients with a manic predominant polarity, but should probably not be recommended to patients who have a depressive predominant polarity. Given that many patients living with bipolar disorder have a clear tendency toward one pole or another, there is a need for longitudinal course specifiers to help HCPs to better understand a particular patient and make informed decisions regarding treatment choices and approach.^17^ However, it is important to highlight that second‐ or third‐generation LAIs should be the preferable option because first‐generation LAIs may worsen symptoms of depression,^60^ which, in turn, may result in non‐adherence.

### 3.2 The formulation

While LAIs offer convenience in the form of longer administration intervals, the injectable formulation does result in several potential barriers to early initiation and long‐term adherence, including accessibility challenges (availability, infrastructure, and maintenance logistics) and HCP and patient attitudinal barriers.

#### 3.2.1 Accessibility challenges—availability/reimbursement

Guidelines are important for HCP awareness regarding the suitability of a treatment and for making informed treatment decisions. Although some guidelines, such as the International College of Neuropsychopharmacology guidelines,^35^ do consider the use of LAIs for BP‐I, they do not yet have a major role in other international guidelines for BP‐I, including the Canadian Network for Mood and Anxiety Treatments guidelines,^34^ the British Association for Psychopharmacology guidelines,^49^ the World Federation of Societies of Biological Psychiatry guidelines,^61^ and the Royal Australian and New Zealand College of Psychiatrists guidelines.^62^ It is important to note that international guidelines may be made obsolete early on if the latest findings are available soon after publication. Communication between HCPs and governments is crucial to highlight the benefits of LAIs for BP‐I in the public and private health sectors. If a medication is licensed and recommended by experts worldwide, it may be more likely to be accepted into reimbursement schemes and provide the confidence needed for HCPs to prescribe.

#### 3.2.2 Accessibility challenges—infrastructure and maintenance logistics

The requirement for additional resources and infrastructure for LAI use, compared to oral medications, can be a key accessibility barrier. Traditionally, healthcare facilities (i.e., clinics and hospitals) with staff experienced in caring for patients with psychiatric disorders have been required to administer LAIs; such facilities should exist within manageable distances of the patient and be open at convenient times. Adequate staffing levels are also critical. In regions where these needs are not being met, especially in lower‐resourced settings, the likelihood of non‐attendance is increased. While home visits to administer injections may represent an alternative approach,^63^ these can also be limited by low staffing levels or other barriers. Pharmacists have increasingly been employed to deliver LAIs, which represents an important advancement in access because pharmacies are among the most ubiquitous and accessible healthcare settings.^64^

The post‐COVID‐19 healthcare landscape may also be a barrier to clinicians adopting new treatment strategies.^21^ The administration of LAIs was suspended in some areas during the COVID‐19 pandemic because it was considered an “elective” procedure.^65^ The suspension prompted the American Psychiatric Association to issue specific COVID‐19 pandemic guidance on the administration of LAIs, encouraging clinics, hospitals, and other medical facilities to include the ongoing use of LAIs for patients with high‐risk chronic illness as a necessary procedure, noting that treatment withdrawal would likely increase the risk of physical and psychiatric collapse.^66^ However, as these guidelines only referred to severe cases, this may exacerbate the preconceived notion that LAIs can be used only for patients with the most severe disease.

#### 3.2.3 Attitudinal barriers

A perception that oral antipsychotics represent the best treatment for all patients leads to this option being well prescribed and may hinder the widespread use of LAIs, with physician recommendations influencing patients' decisions about treatment options.^67^ This perception is likely due to clinicians having extensive experience with oral treatments, especially when compared to LAIs.

Treatment adherence may be influenced by a fear of needles and/or a patient's negative experiences in an emergency setting. An injectable medication could be perceived as invasive, invoking feelings of punishment instead of help and relief.^68^, ^69^, ^70^ However, it has been shown that patients' concerns regarding the use of LAIs for schizophrenia or schizoaffective disorder may be overestimated.^71^ Perceptions are similar for BP‐I. Despite previously hearing a negative presentation regarding LAIs, a subsequent positive presentation accompanied by additional information has resulted in increased patient willingness to try LAI therapy.^72^ Looking to other examples of patient engagement and education, studies have reported that for patients living with bipolar disorder, negative illness perceptions are associated with more severe mood symptoms.^73^ Interventions, such as education and engagement, designed to enhance favorable illness perception and reduce unfavorable perceptions, may improve mood outcomes, particularly when patients adopt regular social rhythms.^73^ This evidence suggests that by engaging positively with the patient around their condition and providing all the available treatment options, outcomes may be improved.^74^

#### 3.2.4 Formulation benefits

**FIGURE 1:**
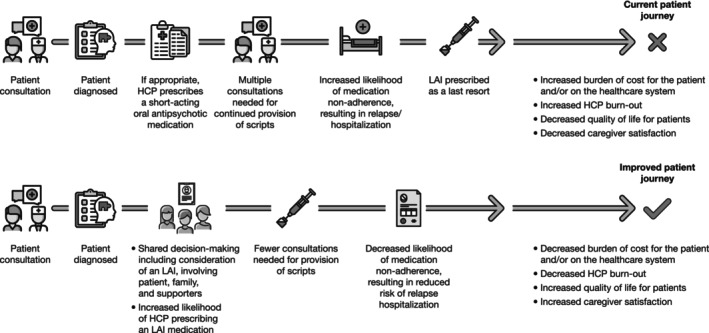
Typical patient journey compared to the ideal LAI patient journey. HCP, healthcare provider; LAI, long‐acting injectable antipsychotic.

Once administered, a major benefit of LAIs is their increased duration of effect, extending to weeks or months. In BP‐I, LAIs are associated with improved treatment adherence,^75^ better patient outcomes (e.g., reduced relapse and hospitalization),^41^, ^46^, ^47^ and a reduced burden on healthcare systems,^25^ compared to oral antipsychotics (Figure 1).

#### 3.2.5 The future and next steps

Despite the historically low usage, LAI use does appear to be increasing, as the prescription rate for bipolar mania has risen significantly from 2.20% in 2006 to 11.58% in 2018.^46^ It is important that HCPs inform patients of all available treatment options as early as possible in the course of illness, as some patients may find LAIs to be more convenient than having to take oral medication daily. Some patients might respond negatively to a failure to disclose all possible options, which may be viewed as paternalistic. In some countries (e.g., Australia and the United States), where nurse practitioners are often the main point of contact or the primary caregiver in community mental healthcare settings, LAIs are more likely to be used by nurse practitioners than psychiatrists.^76^, ^77^ The expanded practice of some pharmacists delivering LAIs further supports the importance of widely disseminating knowledge across all allied health professions.^64^ This is critical so that all providers/professionals involved in the care of patients living with BP‐I are aligned on the information provided to patients and their families.

### 3.3 The societal impact

**FIGURE 2:**
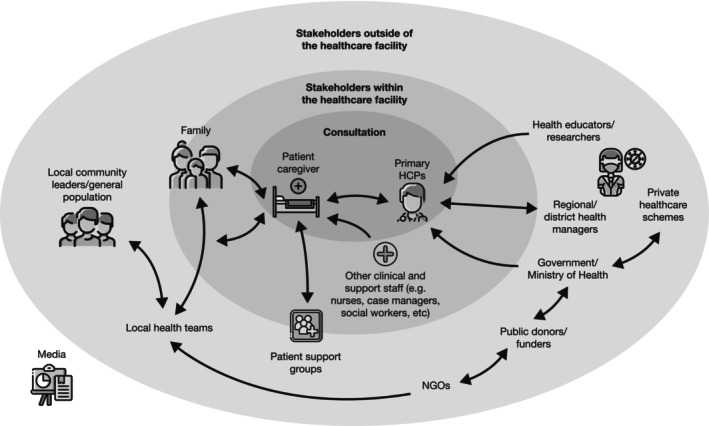
Relationships between stakeholders. HCP, healthcare provider; NGO, non‐governmental organization.

To achieve optimal outcomes for patients living with BP‐I, it is critical to involve all stakeholders who are part of the patient journey, such as HCPs, patients, and their supporters, in an early shared decision‐making process (Figure 2). As described previously, early disease represents an excellent opportunity to begin this process and pivot away from traditional clinical practices. Consistent accessibility to psychiatrists varies greatly worldwide, as emphasized by the COVID‐19 pandemic, highlighting the need for treatments that decrease dependence on healthcare systems and the need for the stability that a longer‐acting antipsychotic may provide.^21^

More data are required to assess the potential impact of LAIs on BP‐I and to facilitate widespread acceptance of LAIs. Clinical and database studies could aid in bridging the existing knowledge gaps and, subsequently, provide HCPs and clinics with first‐hand experience from clinicians who prescribed LAIs for BP‐I earlier in the course of illness. Although naturalistic randomized clinical trials (RCTs) can be complex to organize and complete, they are invaluable because they do not require blinding and better reflect real clinical practice while still being more controlled than real‐world studies. For instance, trials to compare LAIs with lithium (the “gold standard”), especially in relation to the dangers of lithium discontinuation, are highly desirable if current clinical practice is to be altered. The PRELAPSE study in early‐phase schizophrenia was a well‐designed naturalistic RCT involving 489 participants in which the use of long‐acting injectable aripiprazole monohydrate was associated with a significant delay in time to first hospitalization,^40^ and a similar study for BP‐I would be welcomed. Databases represent an alternative source of valuable information and can provide rapid insight with real‐world data at a relatively low cost. They may also provide information on the reduction of polypharmacy burden, an important consideration in the BP‐I population.^78^, ^79^ Large databases and observational studies, in particular, are invaluable in achieving substantial sample sizes to assess real‐world effectiveness.

The concomitant use of LAIs and mood stabilizers should also be adequately assessed, offering insights to help ease burden on patients and healthcare systems. Preliminary evidence has shown that second‐generation LAIs used for BP‐I, such as adjunctive risperidone, can protect against manic relapses/recurrences in patients living with BP‐I,^80^ but not against depression, and so combination with appropriate other treatments (e.g., lamotrigine) should be investigated. Most RCTs of combination pharmacotherapy focus on the benefit of pairing a mood stabilizer with a second‐ or third‐generation antipsychotic for the prevention of either acute mania or relapse;^81^ however, in clinical practice, patients living with bipolar disorder often take more elaborate combinations of mood stabilizers, antipsychotics, antidepressants, and other psychotropic medications for indefinite periods that do not necessarily follow a strategic or logical course. Thus, tracking of outcomes through both clinical and database studies is of great importance so that HCPs can devise regimens that are complementary, non‐redundant, purposeful, and evidence based.^81^

Finally, while clinical trials and other research methods are important in helping raise awareness of LAIs for BP‐I, additional avenues could include increased education for HCPs about the benefits of LAIs (online courses and congress presentations), scientific publications (including reviews and guidelines), and direct patient education.

## 4 OVERALL CONCLUSIONS AND EXPERT RECOMMENDATIONS

This consensus discussed the benefits of LAIs in the management of BP‐I, particularly during early disease, and identified barriers to overcome, while providing potential solutions to aid key stakeholders in making informed treatment decisions. The main consensus recommendations were as follows.

### 4.1 Consensus recommendation 1

It is important to move away from the preconceived notion that LAIs can be used only for patients living with BP‐I with the most severe disease, and communication of potential early benefits is key to avoid perpetuating stigmatization of patients.

### 4.2 Consensus recommendation 2

If antipsychotic therapy is included in the pharmacologic treatment plan, LAIs should be initiated as early as possible in the disease course, ideally at the first manic episode, to aid in improving long‐term outcomes.

### 4.3 Consensus recommendation 3

A collaborative focus on the direct involvement of all stakeholders in the decision‐making process should support a more positive patient experience, better care quality, and improved health outcomes (e.g., reduced relapse and hospitalization, and reduced burden on healthcare systems).

### 4.4 Consensus recommendation 4

With careful outcome tracking and a systematic approach to medication changes, logical and strategic combination pharmacotherapy can be designed to minimize unnecessary exposure to redundant, ineffective, or otherwise superfluous psychotropic agents.

### 4.5 Consensus recommendation 5

The generation of additional evidence for the use of LAIs early in the disease course of BP‐I is critical to challenge existing attitudes and support any recommendations for use in regional practice guidelines and healthcare systems.

## AUTHOR CONTRIBUTIONS

All authors significantly contributed to the conception, design, acquisition, analysis or interpretation of the data for the work. All authors collectively and critically reviewed the manuscript, granting final approval. Each author has assumed accountability for all aspects of the work, committing to address and resolve any questions related to its accuracy or integrity.

## FUNDING INFORMATION

The round table meeting leading to this expert consensus was funded and supported by Otsuka Lundbeck Alliance.

## CONFLICT OF INTEREST STATEMENT

E.V. has received grants and served as a consultant, advisor, or CME speaker for AB‐Biotics, Abbott, AbbVie, Aimentia, Angelini, Biogen, Biohaven, Boehringer Ingelheim, Casen‐Recordati, Celon, Compass, Dainippon Sumitomo, Ethypharm, Ferrer, Gedeon Richter, GH Research, GSK, Idorsia, Janssen, Lundbeck, Novartis, Organon, Otsuka, Rovi, Sage, Sanofi‐Aventis, Sunovion, Takeda, and Viatris. M.T. was an employee of Lilly (1997–2008) and has received honoraria from or consulted for Abbott, AbbVie, Alkermes, AstraZeneca, Atai Life Sciences, Biohaven Pharmaceuticals, Bristol Myers Squibb, Elan, Intra‐Cellular Therapies, Johnson & Johnson, Lilly, Lundbeck, Merck, Minerva, Neurocrine Biosciences, NeuroRX, NoemaPharma, Otsuka, Pfizer, Rapport Neurosciences, Roche, Sunovion, and Teva; his spouse was a full‐time employee at Lilly (1998–2013). D.M. is on advisory boards/speaker panels for AbbVie, Janssen Ortho, Lundbeck, Otsuka, and Sunovion. L.V.K. has been a consultant for Lundbeck and Teva. M.S. has research grants from Centers for Disease Control and Prevention, International Society for Bipolar Disorders, National Institutes of Health, Patient‐Centered Outcomes Research Institute; consultancy for Alkermes, Janssen, Lundbeck, Neurelis, Otsuka, Teva; royalties from Johns Hopkins University Press, Oxford University Press, Springer Press, UpToDate; and compensation for CME activities from American Academy of Child and Adolescent Psychiatry, American Epilepsy Society, American Physician Institute, Clinical Care Options, Neurocrine, and Psychopharmacology Institute. R.S.M. has received research grant support from CIHR/GACD/NSFC and the Milken Institute; speaker/consultation fees from AbbVie, Alkermes, Atai Life Sciences, Axsome, Bausch Health, Biogen, Boehringer Ingelheim, Eisai, Intra‐Cellular Therapies, Janssen, Kris, Lundbeck, Mitsubishi Tanabe, Neumora Therapeutics, Neurawell, Neurocrine, New Bridge Pharmaceuticals, Novo Nordisk, Otsuka, Pfizer, Purdue, Sage, Sanofi, Sunovion, Takeda, Viatris; and is a CEO of Braxia Scientific Corp.

## Data Availability

Data sharing is not applicable to this article as no new data were created or analyzed in this study.
